# Diagnostic accuracy of automated ACR BI-RADS breast density classification using deep convolutional neural networks

**DOI:** 10.1007/s00330-023-09474-7

**Published:** 2023-03-01

**Authors:** Raphael Sexauer, Patryk Hejduk, Karol Borkowski, Carlotta Ruppert, Thomas Weikert, Sophie Dellas, Noemi Schmidt

**Affiliations:** 1 Department of Radiology and Nuclear Medicine, University Hospital Basel, Petersgraben 4, CH-4031 Basel, Switzerland; 2 Institute of Diagnostic and Interventional Radiology, University Hospital Zurich, Rämistrasse 100, CH-8091 Zurich, Switzerland

**Keywords:** Deep learning, Breast density, Risk factors, Mammography, Breast neoplasms

## Abstract

**Objectives:**

High breast density is a well-known risk factor for breast cancer. This study aimed to develop and adapt two (MLO, CC) deep convolutional neural networks (DCNN) for automatic breast density classification on synthetic 2D tomosynthesis reconstructions.

**Methods:**

In total, 4605 synthetic 2D images (1665 patients, age: 57 ± 37 years) were labeled according to the ACR (American College of Radiology) density (A-D). Two DCNNs with 11 convolutional layers and 3 fully connected layers each, were trained with 70% of the data, whereas 20% was used for validation. The remaining 10% were used as a separate test dataset with 460 images (380 patients). All mammograms in the test dataset were read blinded by two radiologists (reader 1 with two and reader 2 with 11 years of dedicated mammographic experience in breast imaging), and the consensus was formed as the reference standard. The inter- and intra-reader reliabilities were assessed by calculating Cohen’s kappa coefficients, and diagnostic accuracy measures of automated classification were evaluated.

**Results:**

The two models for MLO and CC projections had a mean sensitivity of 80.4% (95%-CI 72.2–86.9), a specificity of 89.3% (95%-CI 85.4–92.3), and an accuracy of 89.6% (95%-CI 88.1–90.9) in the differentiation between ACR A/B and ACR C/D. DCNN versus human and inter-reader agreement were both “substantial” (Cohen’s kappa: 0.61 versus 0.63).

**Conclusion:**

The DCNN allows accurate, standardized, and observer-independent classification of breast density based on the ACR BI-RADS system.

**Key Points:**

• *A DCNN performs on par with human experts in breast density assessment for synthetic 2D tomosynthesis reconstructions*.

• *The proposed technique may be useful for accurate, standardized, and observer-independent breast density evaluation of tomosynthesis*.

## Introduction

Breast cancer is the most frequently diagnosed cancer among females with an incidence of 12.3% in the normal population [1]. Breast density, defined as the amount of fibroglandular tissue relative to fatty tissue, correlates with increasing breast cancer risk [2, 3]. Cancer detection rate is inversely related to density, since carcinomas, which present as masses, in particular without microcalcifications, can be masked [4].

Digital breast tomosynthesis (DBT) as a quasi-3D modality is increasingly used to determine breast density and for cancer detection, as it decreases the superimposition of breast tissue [5], with high diagnostic accuracy to detect breast cancer with an AUC, sensitivity, and specificity of 0.95, 0.90, and 0.90, respectively [6]. To improve breast density reporting, the ACR BI-RADS Atlas 5th Edition classifies breast density into A-D: A (“almost entirely fatty”), B (“scattered areas of fibroglandular density”), C (“heterogeneously dense breasts, which may obscure small masses”), and D (“extremely dense breasts, which lower the sensitivity of mammography”). The recently published European Society of Breast Imaging (EUSOBI) recommends contrast-enhanced breast MRI in women of 50–70 years of age with extremely dense breasts in routine mammography screening as supplemental screening for breast cancer mortality reduction and advocates to inform women about their breast densities [7].

However, breast density reporting is still prone to inter- and intra-reader variability with a broad range of reported kappa-values in the literature [8]. To address this need for reproducibility and simpler clinical implementation, deep learning can reduce inter-reader variability [9], and improve standardized reporting [10].

### Study objectives and hypotheses

This study aimed to develop a deep convolutional neural network (DCNN) for the automatic classification of breast density in synthetic 2D reconstructions of digital breast tomosynthesis according to the American College of Radiology Breast Imaging Reporting and Data System (ACR BI-RADS) Atlas.

## Methods

The local ethics committee approved this retrospective study (Project ID: 2021–01472).

### Study population

The retrospective selection was made of all female patients who underwent diagnostic exams or opportunistic screening exams using synthetic 2D tomosynthesis between February 2018 and February 2020. 2D FFDM (full-field digital mammography) mammograms produced as a part of the regional screening program, 2D magnification images, and 2D images used in stereotactic biopsies were disregarded. Patients with breast implants, DIEP reconstructions, tomosynthesis images taken during stereotactic biopsies, and ML views were excluded, as were patients without consent and those who had invalid imaging data.

### Tomosynthesis acquisition parameters

All DBT examinations were performed with a wide-angle DBT system (Selenia**®** Dimensions**®** Mammography System, Hologic**®**) and synthetic two-dimensional images were generated by using the vendor’s reconstruction software (C-View™ software). The synthetic 2D images commonly replace the FFDM for density assessment during the acquisition of a tomosynthesis in order to reduce radiation dose.

### Data preparation

The only model inputs used were synthetic 2D images. Since we had no intention of volumetric determination, we did not employ the single tomosynthesis slices. All images were resized from their initial dimensions of 3072 × 2816 pixels to 224 × 224 pixels and images from the right craniocaudal (RCC) and right mediolateral oblique (RMLO) orientations have been flipped horizontally to position the breast on the same side as the according image. Data augmentation was applied to the training dataset with the TensorFlow (Google LLC) ImageDataGenerator class by random vertical shifts, zooming, and rotating images to improve the generalization of the network (height_shift_range = 0.1; zoom_range = 0.1; rotation_range = 10). A class weighting method was used to achieve an equal distribution of breast densities for the training phase. All eligible mammograms were randomly split into three datasets: training (70%), validation (20%), and test dataset (10%).

### Reference standard

A board-certified radiologist with 2 years of experience in mammographic imaging (= reader 1) classified all eligible mammograms according to the BI-RADS density description in A-D on a diagnostic monitor (EIZO RX350, resolution: 1536 × 2048, EIZO).

To assess inter- and intra-reader variability, all cases in the test data set were rated by reader 1 twice, one month apart (reader 1A and reader 1B), and a radiologist with 11 years of experience in mammographic imaging (reader 2). Both readers were blinded to ACR densities (AI and prior examinations). Disagreement was solved by a consensus discussion between reader 1 and reader 2. The consensus was used to evaluate the model’s performance. In addition, the density classifications were exported from the initial radiological reports. For external validation, we used a data set from the University Hospital Zurich with a consensus reading of 67 mammograms by two board-certified radiologists (7 and 12 years of experience).

### Model

Two DCNN models were used for the classification of MLO and CC views. Each model consisted of 11 convolutional layers, 3 fully connected layers, 5 downsampling max-0 layers, and 2 dense layers with a Rectified Linear Unit (ReLU) activation function. A 50% dropout was used to reduce overfitting. A softmax activation function was used to obtain the final weights from a model. The models were trained with the Adam optimizer and Categorical Cross-Entropy loss function. Batches of 16 images have been fed to the network. Training hyperparameters have been fine-tuned based on the maximization of validation accuracy during training, whereby the number of epochs was determined by training accuracy exceeding 5% of the validation accuracy: The models have been trained for 200 epochs with a learning rate of 1.0 × 10^–5^. Models were trained using a TensorFlow 2.0 platform on an Nvidia 1080 GTX GPU running on a Ubuntu 16.04 OS. In the case of different predictions for mediolateral oblique (MLO) and craniocaudal (CC), according to the ACR-BIRADS catalogue, the higher density prediction was assigned as the overall density.

### Statistical analysis

Cohen’s kappa (κ) coefficients were used to assess inter- and intra-reader agreement of ACR-density classifications between both readers and the models, as well as the reference standard/consensus. By convention, values of < 0.0, 0.00–0.20, 0.21–0.40, 0.41–0.60, 0.61–0.80, and 0.81–1.00 are, respectively, indicative of poor, slight, fair, moderate, substantial, and almost perfect agreement [11]. Sensitivity, specificity, negative predictive value (NPV), and positive predictive value (PPV) are used to evaluate the diagnostic performance of the DCNN compared to the reference standard/consensus. The multiple classification problem of the test dataset with four density categories (i.e., A, B, C, and D) was translated into four binary classification problems (i.e., A vs all; B vs all; C vs all; and D vs all). For each binary classification, diagnostic accuracy measures of b-density software were calculated independently. All tests were two-tailed, and *p* values of < 0.05 were considered significant. All statistical analysis was performed with R 4.0.5 (R Core Team, R Foundation for Statistical Computing) and refers to the test dataset unless otherwise specified.

### Breast density assessment

Breast density based on the mammographic appearance was automatically assessed from the synthetic 2D images CC and MLO projections using the commercially available “b-box” AI platform (Medical device type IIa, CE 0297) by b-rayZ AG (version 1.1.0, b-rayZ AG) in which the previously trained models were implemented. The overall density classification was based on the highest probability for one of the four categories A to D.

## Results

### Study population

**Fig. 1:**
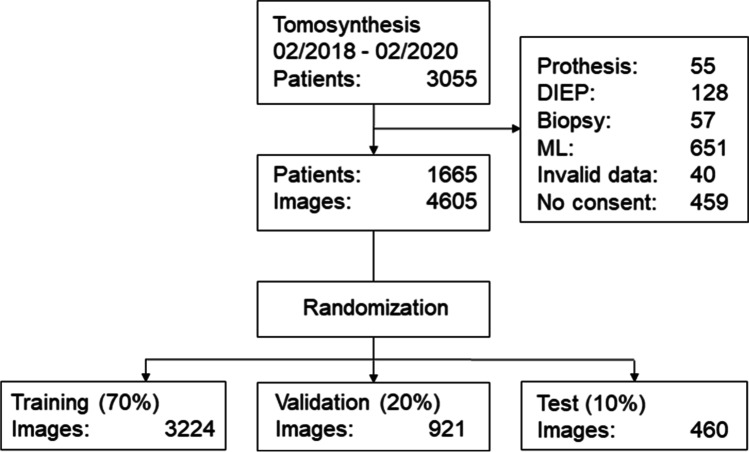
Study flow diagram

A total of 1665 female patients with a total of 4605 mammograms were included in this study (see Fig. 1). Of these, 380 were used for the test dataset (460 images, mean age: 58.9, range: 26–90). The distribution of ACR density in the test data set is ACR A: 10.9% (*n* = 50), B: 62.4% (*n* = 287), C: 23.0% (*n* = 106), and D: 3.7% (*n* = 17).

There were no significant differences between the training, validation, and test datasets regarding age (*p* > 0.50) and density distribution (*p* > 0.99).

### Inter-reader agreement

For combined MLO and CC projections, the intra-reader agreement for reader 1 was “almost perfect” [14] with a kappa score of 0.82 (*p* ≤ 0.001). There was substantial agreement between both readers (Cohens kappa 0.63, *p* ≤ 0.001) with the same BI-RADS class in 370 out of 460 cases. The consensus was formed in 90 cases (19.6%) by discussion.

### Model performance

**Fig. 2:**
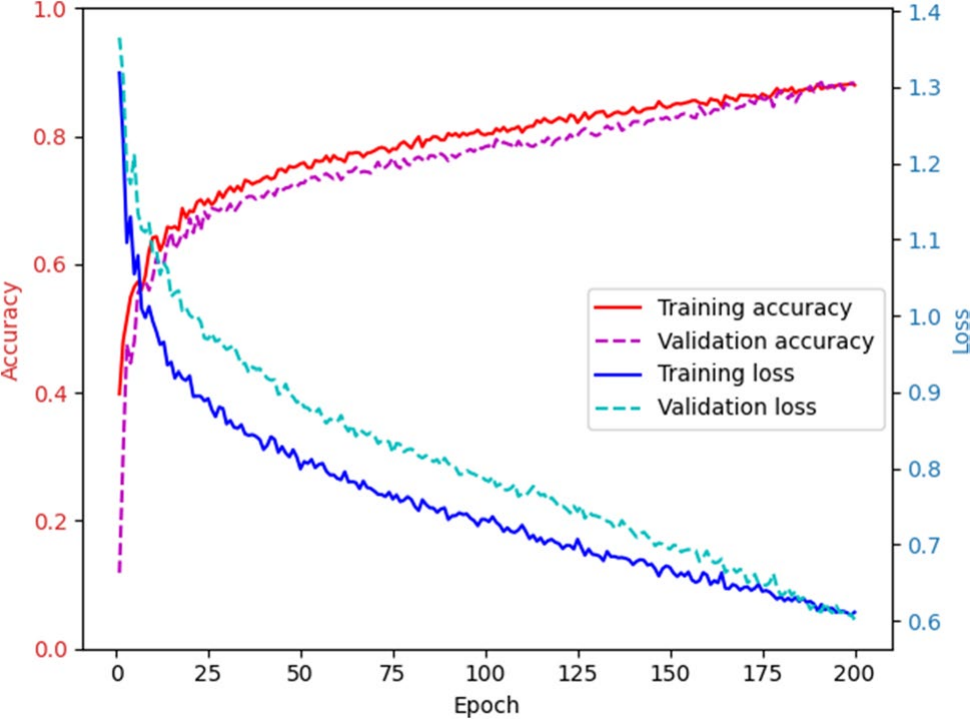
Maximization of diagnostic accuracy using the validation dataset by adjusting training hyperparameters over 200 epochs. Red shows the training accuracy; pink, the validation accuracy; blue, the training loss; and light blue, the validation loss

**Fig. 3:**
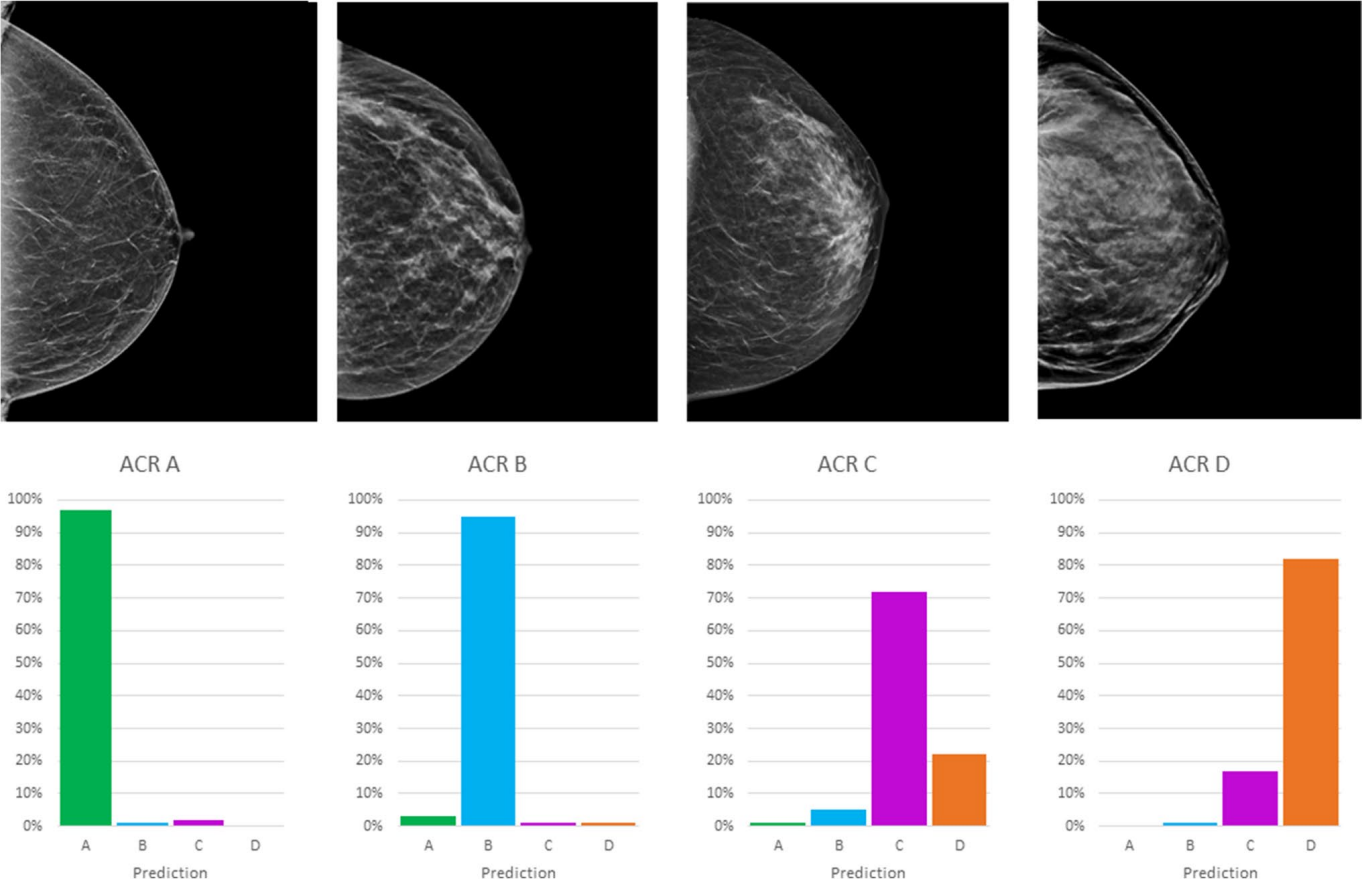
Diagnostic accuracy of ACR density A-D classification with representative examples of each based on the test data set for the CC projections

**Fig. 4:**
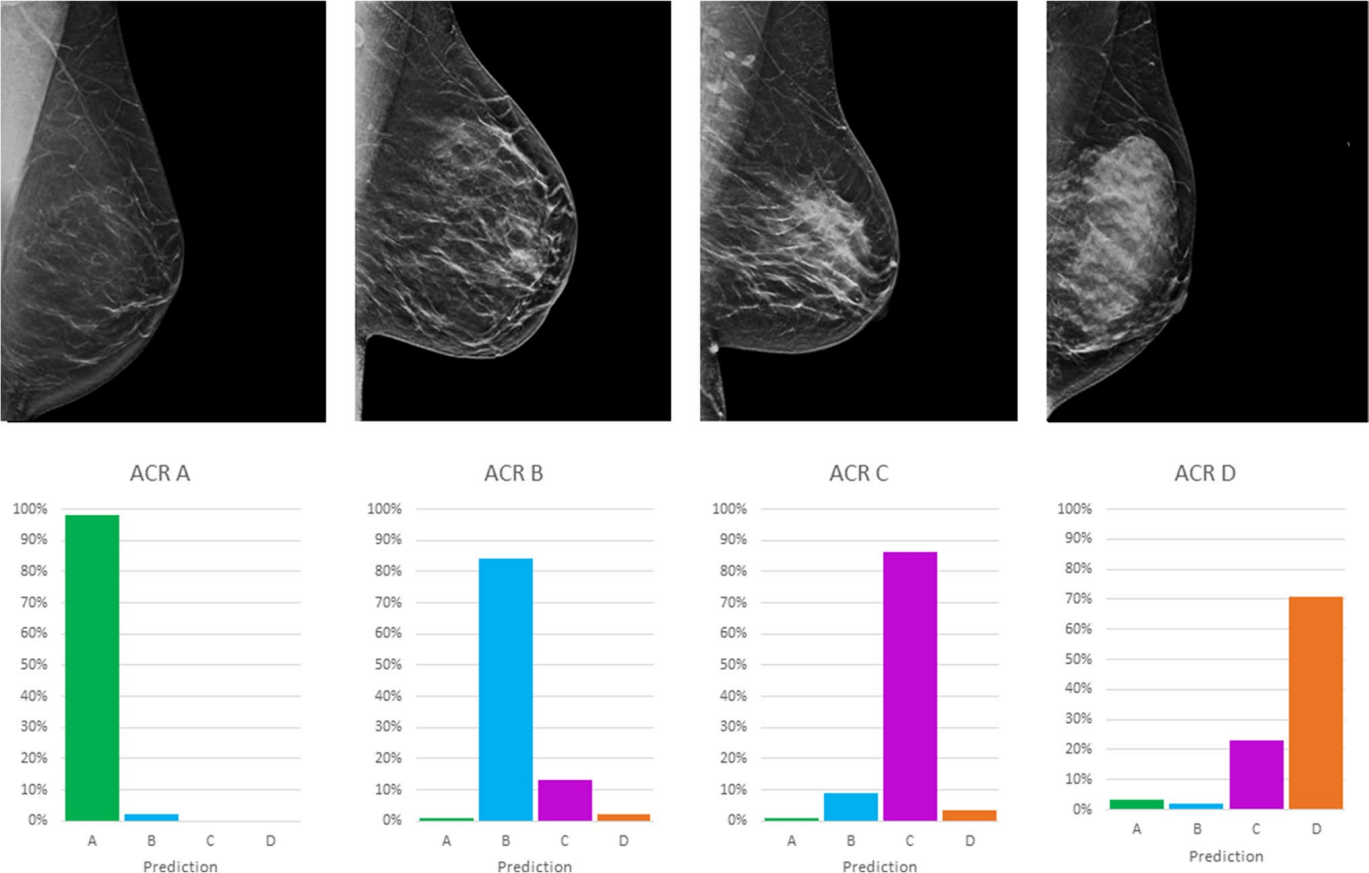
Diagnostic accuracy of ACR density A-D classification with representative examples of each based on the test data set for the MLO projections

As compared to the consensus decision of the two radiologists, the DCNNs showed an overall sensitivity of 79.1% (95%-confidence interval (CI): 75.1–82.8), a specificity of 93.0% (95%-CI: 91.6–94.3), a PPV of 79.1% (95%-CI: 75.7–82.2), an NPV of 93.0% (95%-CI: 91.8–94.1), and an accuracy of 89.6% (95%-CI: 88.1–90.9) based on the test dataset. This corresponded to an improvement of accuracy over the initial radiological reports (84.2%, 95%-CI: 82.5–85.9%) with a sensitivity of 68.9% (95%-CI: 64.4–73.0) and specificity of 89.5% (95%-CI: 89.4–87.7). The training/validation accuracy is shown in Fig. 2, whereas Figs. 3 and 4 summarize the diagnostic accuracies for CC and MLO, respectively. Classification of ACR density B showed the highest sensitivity (86.8%), whereas classification of ACR density A and D showed the highest specificities (99.5% and 99.1%). If only density classes A/B and C/D are considered, the sensitivity is 80.4% (95%-CI: 72.2–86.9), the specificity 89.3% (95%-CI: 85.4–92.3), the PPV 73.3% (95%-CI: 64.9–80.4), and the NPV 92.6% (95%-CI: 89.1–95.1). For both projections, no significant differences in diagnostic accuracy were found between the two readers and the DCNN. The diagnostic accuracy measures for density classification are summarized in Table 1. We found a substantial agreement between the consensus and DCNN (kappa 0.61, *p* < 0.001).

**Table 1:** Cross tabulation, sensitivity, specificity, positive predictive value (PPV), and negative predictive value and their precision (95% confidence intervals) based on the test data set

	ACR A	ACR B	ACR C	ACR D
TP	24	249	79	12
TN	408	123	314	439
FP	2	50	40	4
FN	26	38	27	5
Sensitivity (95%-CI)	48.0 (33.9–62.6)	86.8 (82.2–90.5)	74.5 (65.1–82.5)	70.6 (44.0–89.7)
Specificity (95%-CI)	99.5 (98.1–99.9)	71.1 (63.6–77.7)	88.7 (84.9–91.8)	99.1 (97.7–99.8)
PPV (95%-CI)	92.3 (74.5–98.0)	83.3 (79.7–86.3)	66.4 (59.1–73.0)	75.0 (51.9–89.3)
NPV (95%-CI)	94.0 (91.2–96.0)	76.4 (70.3–81.5)	92.1 (89.3–94.2)	98.9 (97.7–99.5)
Accuracy (95%-CI)	93.9 (91.3–95.9)	80.9 (77.0–84.4)	85.4 (81.9–88.5)	98.0 (96.3–99.1)
Prevalence (95%-CI)	10.9 (8.2–14.1)	62.4 (57.8–66.8)	23.0 (19.3–27.2)	3.7 (2.2–6.0)

**Fig. 5:**
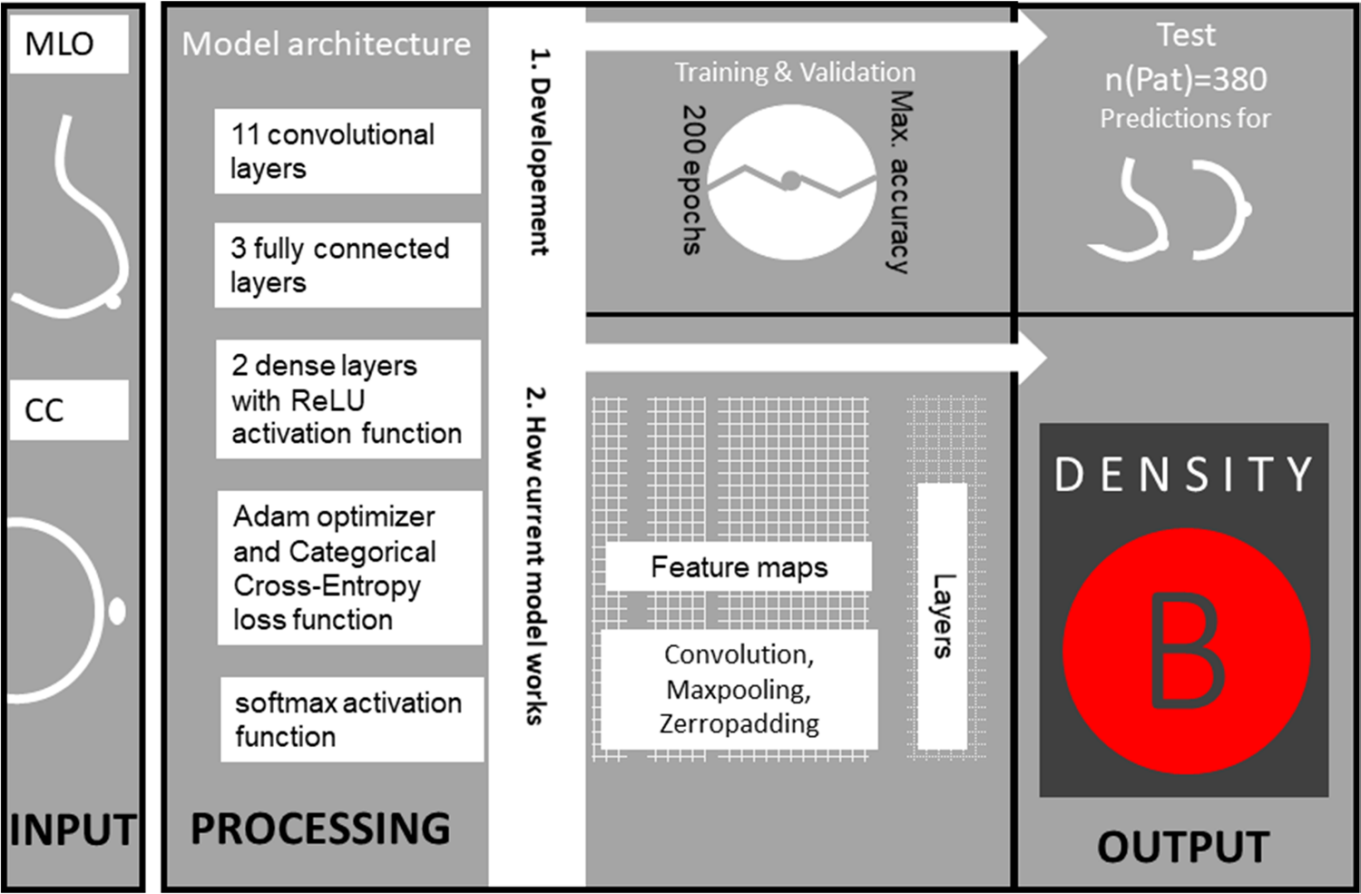
Schematic pattern of the applied multilayered deep convolutional neural network (dCNN), containing 11 convolutional layers 3 fully connected layers, 5 downsampling max-0 layers, and 2 dense layers with a Rectified Linear Unit (ReLU) activation function

In the external validation, the models achieved a sensitivity, specificity, and accuracy of 68.7% (95%-CI: 56.2–79.4), 89.6% (95%-CI: 84.5–93.4), and 84.3% (95%-CI: 79.4–88.5), respectively. In summary, Fig. 5 shows a simplified scheme of model development, current processing, and the user interface using the example of an ACR B dense breast.

## Discussion

In the present study, we propose an automatic approach for the determination of breast density for synthetic 2D reconstructions in digital breast tomosynthesis according to the ACR BI-RADS catalog using a machine learning algorithm based on a deep convolutional neural network. The DCNN was trained with 3224 synthetic 2D images. For the implemented DCNN, an optimal number of 200 epochs was determined, reaching an accuracy of 89.6%.

While BI-RADS-based reporting is routinely used in breast imaging, deep convolution networks hold promise for a variety of AI-driven standardizations [12]. As we already showed, Deep convolutional networks are capable of FFDM BI-RADS breast density prediction [13] and a high level of agreement in radiological assessment exists for FFDM [14] the present study proves the suitability of our network for density assessment of synthetic 2D tomosynthesis reconstructions. Herein, we found a substantial inter-reader agreement between the four-category ACR BI-RADS categories, which are known to be observer-dependent with kappa-values ranging between 0.43 and 0.89 [15–17].

As the feasibility of automated prediction has been shown on smaller population numbers [16, 18, 19], several models were developed previously for both FFDM only [20–23] or a mixture of FFDM and DBT images [17, 22].

However, there is large heterogeneity regarding the reference standard: Le Boulc’h M et al used only CC images for the density classification [19], and Pahwa S et al classified each breast separately, which both do not correspond to clinical routine [23]. Some authors used the old BI-RADS density classification 1–4, volumetric classifications [20], visual analogue scale (VAS) scale from 0 to 100% [21], or a binary classification “dense versus not dense” [22]. Some were partially interpreted by non-radiologists [21] or used FFDM as training input [24]. In contrast to conventional automated volumetric breast density assessments, which are prone to false positives due to minor dense regions, the models are trained with the visual impression by experienced radiologists, which is the most used and therefore best reference standard suited for breast density. We used all densities of the current BI-RADS edition and proposed a well-rounded differentiation of the density categories, taking into account not only accuracy [24, 25] but also the sensitivity, specificity, PPV, NPV, TP, and FP and improved comparability by standardized reporting using the STARD 2015 guideline for diagnostic accuracy studies [26].

In accordance with our results, Mohamed et al also recently showed that a DCNN algorithm can discriminate between categories B and C with an accuracy of 94% as compared to the radiological reports of the local institution [27]. This holds also true for tomosynthesis. Diagnostic accuracy, agreement, and reliability were similar to a recent study by Magni et al, with a smaller study population (577 patients, 1144 images) but high-quality reference standard by 7 board-certified radiologists [28].

Interestingly, Gastounioti et al showed a stronger association between breast cancer risk and density with DBT compared to digital mammography [29]. However, the study population was relatively small (*n* = 132).

The adaptability of the network, as shown here for 2D synthetic tomosynthesis reconstructions, could also be of interest for both different classification tasks and other modalities like ultrasound MRI, contrast-enhanced mammography, or CT [30–34] which have become increasingly important due to advances in acquisition in recent years [35, 36].

Density measurement serves to estimate breast cancer risk and inform patients. It could help optimize individual cancer detection regimens and could guide recommendations for supplemental or alternative screening options such as ultrasound or MRI. This model architecture which enables quick predictions even with limited hardware performance was designed to fulfill clinical requirements.

### Study limitations

This is a single-center study with a DTB of one vendor and the external validation dataset was relatively small. Since this is one of the first studies on the use of convolutional neural networks for classifying BI-RADS density based on synthetic two-dimensional images generated from tomosynthesis images, a comparison with other pre-trained models could unfortunately not be carried out. The exclusion of postoperative exams with DIEP reconstruction or breast implants might limit the use of the models in the postoperative setting. Finally, the reference standard for tomosynthesis is primarily based on only two experienced radiologists, who, however, have a subspecialty in breast imaging with a total of 13 years of experience.

## Conclusion

In conclusion, a DCNN trained on *n* = 3224 DBTs allowed an accurate, standardized, and observer-independent classification of breast density according to the ACR BI-RADS catalog. The implementation of DCNN into the clinical workflow by the “b-box” AI platform could help to improve the diagnostic accuracy and reliability of mammographic breast density assessment in the clinical routine.

## Abbreviations

95% CI: 95% Confidence interval
BI-RADS: Breast Imaging-Reporting and Data System
CC: Craniocaudal
DBT: Digital breast tomosynthesis
DM: Digital mammography
EUSOBI: European Society of Breast Imaging
FFDM: Full-field digital mammography
ML: Mediolateral
MLO: Mediolateral oblique
NPV: Negative predictive value (NPV)
PPV: Positive predictive value (PPV)
ReLU: Rectified linear unit
VAS: Visual Analogue Scale

## Declarations

### Guarantor

The scientific guarantor of this publication is Dr. Noemi Schmidt.

### Statistics and biometry

One of the authors (R. Sexauer) has significant statistical expertise and no complex statistical methods were necessary for this paper.

### Methodology

• retrospective

• experimental

• performed at one institution
